# Supplementary figures and images for: ‘Was my kidney biopsy worth it?’–A qualitative phenomenological study of patient experiences and perceived barriers to kidney biopsy
Source: PLoS One. 2024 Sep 11;19(9):e0310358. doi: 10.1371/journal.pone.0310358 (PMC11389898; doi:10.1371/journal.pone.0310358)

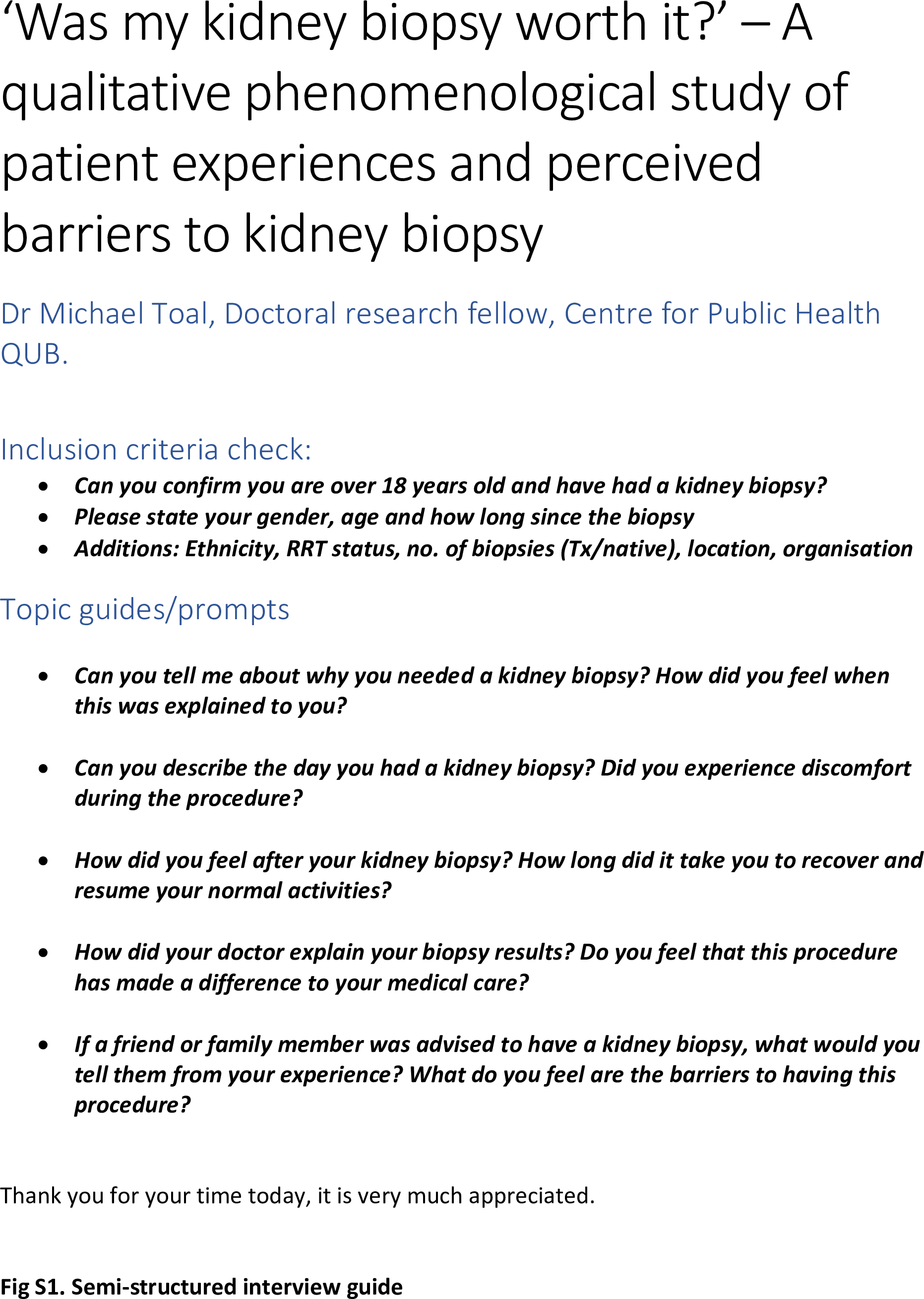

Supplement: S1 Fig — (TIF) [file pone.0310358.s001.tif]
