# Supplementary material for: ‘Was my kidney biopsy worth it?’–A qualitative phenomenological study of patient experiences and perceived barriers to kidney biopsy
Source: PLoS One. 2024 Sep 11;19(9):e0310358. doi: 10.1371/journal.pone.0310358 (PMC11389898; doi:10.1371/journal.pone.0310358)
Supplement: Attachment — Submitted filename: Response to Reviewers_PLoS One_.docx [file pone.0310358.s002.docx]

31 July 2024

Ankur Shah
Academic Editor
PLOS ONE

Dear Dr Shah

PONE-D-23-42064

**"Was my kidney biopsy worth it?" A qualitative phenomenological study of patient experiences and perceived barriers to kidney biopsy**

Thank you for the opportunity to submit a revised manuscript to PLOS One. We really appreciate the time taken by yourself, as the Academic Editor, and the three reviewers to read and constructively critique our manuscript. All of the authors have worked to amend the manuscript incorporating all of the suggested changes.

On behalf of the authors, I have responded to each of the points raised in review. These are detailed below.

I have submitted a marked-up copy of our manuscript highlighting the changes made to the original version (labelled ‘Revised Manuscript with Track Changes’).

I have also submitted an unmarked version of our revised manuscript (labelled ‘Manuscript’).

If there is anything else we can do to assist you consideration of this revised manuscript please do not hesitate to let me know.

Kind regards

Michael Toal

Journal requirements:

When submitting your revision, we need you to address these additional requirements.

1. Please ensure that your manuscript meets PLOS ONE's style requirements, including those for file naming. The PLOS ONE style templates can be found at

<https://journals.plos.org/plosone/s/file?id=wjVg/PLOSOne_formatting_sample_main_body.pdf> and

<https://journals.plos.org/plosone/s/file?id=ba62/PLOSOne_formatting_sample_title_authors_affiliations.pdf>

Thank you for pointing this out. The manuscript has been reformatted in line with the journal requirements.

2. Thank you for stating the following financial disclosure:

“MT is supported by a research award from the Northern Ireland Kidney Research Fund. This organisation was not involved in the design or conduct of this study.

<https://nikidneyresearch.org/>”

Please state what role the funders took in the study.  If the funders had no role, please state: "The funders had no role in study design, data collection and analysis, decision to publish, or preparation of the manuscript."

If this statement is not correct you must amend it as needed.

Please include this amended Role of Funder statement in your cover letter; we will change the online submission form on your behalf.

Thank you for raising this. The wording has been changed in line with your suggestion.

3. In the online submission form you indicate that your data is not available for proprietary reasons and have provided a contact point for accessing this data. Please note that your current contact point is a co-author on this manuscript. According to our Data Policy, the contact point must not be an author on the manuscript and must be an institutional contact, ideally not an individual. Please revise your data statement to a non-author institutional point of contact, such as a data access or ethics committee, and send this to us via return email. Please also include contact information for the third party organization, and please include the full citation of where the data can be found.

You have highlighted an important issue. The statement has now been amended to direct queries to the research ethics officer within the university. Data does not exist on a public repository, so this is not cited, however this could be discussed upon request.

4. Please amend either the title on the online submission form (via Edit Submission) or the title in the manuscript so that they are identical.

This will be updated on the resubmission site.

5. Please include captions for your Supporting Information files at the end of your manuscript, and update any in-text citations to match accordingly. Please see our Supporting Information guidelines for more information: <http://journals.plos.org/plosone/s/supporting-information>.

Thank you, this has been amended appropriately.

[Note: HTML markup is below. Please do not edit.]

Reviewers' comments:

Reviewer's Responses to Questions

**Comments to the Author**

1. Is the manuscript technically sound, and do the data support the conclusions?

The manuscript must describe a technically sound piece of scientific research with data that supports the conclusions. Experiments must have been conducted rigorously, with appropriate controls, replication, and sample sizes. The conclusions must be drawn appropriately based on the data presented.

Reviewer #1: Yes

Reviewer #2: Yes

Reviewer #3: Yes

2. Has the statistical analysis been performed appropriately and rigorously?

Reviewer #1: I Don't Know

Reviewer #2: Yes

Reviewer #3: N/A

3. Have the authors made all data underlying the findings in their manuscript fully available?

The [PLOS Data policy](https://eur02.safelinks.protection.outlook.com/?url=http%3A%2F%2Fwww.plosone.org%2Fstatic%2Fpolicies.action%23sharing&data=05%7C02%7Cmtoal11%40qub.ac.uk%7C23b1790c064e4e37b14c08dca1109f1b%7Ceaab77eab4a549e3a1e8d6dd23a1f286%7C0%7C0%7C638562341221616556%7CUnknown%7CTWFpbGZsb3d8eyJWIjoiMC4wLjAwMDAiLCJQIjoiV2luMzIiLCJBTiI6Ik1haWwiLCJXVCI6Mn0%3D%7C0%7C%7C%7C&sdata=3JH9bEMcmWInK05BVdJ%2FJcfnlBpPW9j%2B2t2M3EfAQDM%3D&reserved=0) requires authors to make all data underlying the findings described in their manuscript fully available without restriction, with rare exception (please refer to the Data Availability Statement in the manuscript PDF file). The data should be provided as part of the manuscript or its supporting information, or deposited to a public repository. For example, in addition to summary statistics, the data points behind means, medians and variance measures should be available. If there are restrictions on publicly sharing data—e.g. participant privacy or use of data from a third party—those must be specified.

Reviewer #1: No

Reviewer #2: Yes

Reviewer #3: Yes

4. Is the manuscript presented in an intelligible fashion and written in standard English?

PLOS ONE does not copyedit accepted manuscripts, so the language in submitted articles must be clear, correct, and unambiguous. Any typographical or grammatical errors should be corrected at revision, so please note any specific errors here.

Reviewer #1: Yes

Reviewer #2: Yes

Reviewer #3: Yes

5. Review Comments to the Author

Please use the space provided to explain your answers to the questions above. You may also include additional comments for the author, including concerns about dual publication, research ethics, or publication ethics. (Please upload your review as an attachment if it exceeds 20,000 characters)

Reviewer #1: This study ist about the the physiological (pain of the procedure...) and psychological aspects (stress, anxiety and felt impact of the treatment course...) of kidney biopsy. The kidney biopsy is an invasive, important diagnostic methode in the nephrology. The study is retrospective and includes 10 partecipants, wich undergoes 23 kidney biopsies. There were used questionnaires. There were included patients with CKD and also kidney transplanted patients.

Up to now there are existing only few studies about the physic and psychological impact in patients. This is a very interestning and import argument. Very simple and clear the introducion and objectives of the study. Methode is simple.

The discussion could be more precise:
Q1A- Interestning is for example the impact of the age: how is the resilience in elder patients, have elder patients more pain? How do you explain the results

A1A: Thank you for raising this point. In our small sample there did not appear to be any clear relationship between age and pain perception. The participant who was youngest at the time of biopsy (14 years) reported minimal pain, as did the oldest participants at time of biopsy (47 years). Although there was no clear correlation between age and perceptions of pain in this study, further research with larger cohorts would be required to try and answer this question.

Q1B- It is also interestning to investigate the results from patients which the native kidney was biopsied versus these which the transplanted kidney was biopsied. Differed the answers of the questionnaires in the groups? How do you explained?

A1B: This is an important point and has also been raised by the other reviewers. Participants who had undergone a kidney transplant before their first biopsy appeared to better tolerate the procedure. There may be several reasons for this relating to the positioning of the patient, their previous experience of invasive procedures in the form of transplant surgery and familiarity with the clinical environment and staff. However this study did not control for these factors, therefore a cautious approach is required in interpretation, as this is beyond the scope of what can be determined by this qualitative approach.

-Q1C It is also interestning to specified in the table the motivation of kidney biopsy: Acute kidney injury (AKI); nephrotic syndrome; autoimmune systemic disease with suspect renal manifestation? How differ the answers in the questionnaire?

A1C: As you rightly point out, there are many situations which may warrant a kidney biopsy, which may precipitate differing experiences for patients. The researchers did not have access to the medical records of participants; therefore these details are incomplete. Participants did volunteer their journey to a kidney biopsy in their own words, which provides some insight into the indications. In this study, several individuals had significant symptoms at the time of the biopsy, such as haemoptysis and leg oedema. Some individuals who were already symptomatic appeared to tolerate the procedure better than asymptomatic persons, however this was not consistent across the entire cohort.

Q1D- Many patients ondergoes multiple kidney biopsy: How is their resilience, pain, felt impact in treatment course? Are differences in confront to patients which ungergoes only one kidney biopsy?

A1D: In this study, 8 out of 10 participants underwent multiple kidney biopsies. In most cases, participants reported that the subsequent biopsies were better tolerated than their first biopsy. However, concluding that subsequent biopsies are better tolerated than the first is beyond the remit of qualitative research.

We would suggest that one reason for this inference could be that participants knew what to expect from the procedure, rather than fearing the unknown. Another important issue was that participants who were undergoing a repeat kidney biopsy were aware of the long clinical observation time and brought resources with them to pass the time, minimising the monotony associated with a long observation period.

Reviewer #2: This is an interesting study by Toal et al describing the patient perspective of undergoing a kidney biopsy using a phenomenological approach.

Overall, the methodology is well described and the core elements of the reporting checklist have been adhered to, which are strengths. I appreciate the context that this is a phenomenological approach, which has some strengths and weaknesses. I believe this would be a meaningful contribution to the literature, but I also have some concerns that I hope the authors could address to strengthen the manuscript.

Major concerns:
Q2A: 1) The context for each theme feels limited, and the representative quotes do not appear to fully justify some components of the Discussion. For example, the authors note in the Discussion section that "the individual who reported the most severe pain also described a significant psychological burden resulting from their kidney disease." However, the quotes in the Results section do not sufficiently describe this psychological burden. It would be helpful to add a few more quotes to the results section, perhaps in a Table or in the main body of the paper. This could help add more context for the general reader for each theme and subtheme outlined in the paper.

A2A: Thank you for raising this point. We have added additional participant quotes to help expand these themes. These additional quotes provide more insights into areas detailed in the discussion. Upon deliberation within the research team, we concluded that it was not constructive to highlight the psychological distress of just one individual, as all participants describe varying degrees of a physical and emotional burden relating to their biopsy and related kidney disease. Therefore this sentence has been removed from the discussion section.

Q2B: 2) I struggled with some aspects of the discussion and results. In particular, I felt that the purpose of the paper was to describe the patient perspective of undergoing a kidney biopsy. However, there were a few sections that appeared to focus more on the experience of living with kidney failure, and were not necessarily related to receiving a kidney biopsy (for example, the notion of having cramps after football, or losing the ability to play rugby). It would be helpful to either clarify this differentiation more explicitly, or mention in the limitations section that some of the experiences described were related to kidney failure and not necessarily to kidney transplantation.

A2B: The reviewer is correct that the main purpose of the paper was to describe the patient perspective of undergoing a kidney biopsy. This is a key issue that has not been well described previously in the literature. The focus of the study was to try and understand the lived experience of having a kidney biopsy, however many participants took this opportunity to also describe, in great detail, their experience of living with kidney disease. Although this is not always directly related to the biopsy, this procedure occurred within the context of their illness (kidney disease), therefore the two are inevitably intertwined through the patient journey. We felt it was important to depict the mental health context in which patients are asked to undergo this invasive procedure, as these individuals’ vulnerability is further tested on top of the physical and emotional toll of their illness. This point has been expanded further in the discussion section.

Q2C: 3) To help the general reader understand the methodology better, please provide more description as to how the qualitative findings were independently verified by an experienced researcher. For example, did the first author and this experienced researcher meet several times to go over the themes and come to consensus? Did the experience qualitative researcher provide asynchronous feedback by email? Did new themes or concepts emerge from these discussions? A fuller description of this approach could help reduce concerns of the undue influence of Dr. Toal's role as a nephrologist who performs kidney biopsy (on a side note, I acknowledge and appreciate that Dr. Toal went to great lengths to describe his position, his own potential biases, and his reflexivity in the methodology section of the paper!)

A2C: This is an important methodological issue, thank you for raising this. We have added further detail within the manuscript of how the stepwise method for Colaizzi’s phenomenological approach was utilised. The first author (MT) identified codes independently in the first instance. Subsequently he met with the experienced qualitative researcher (CMcK) to outline the purpose of the study and relevant methodologies. She then read two sample transcripts, which were the most comprehensive and contrasting and coded these independently, blinded to the initial codes. These new themes were then discussed iteratively to derive new codes, and then expanded to the other transcripts. Each draft of the manuscript was read and amended by CMcK to maintain consistent coding across all participants.

Q2D: 4) The authors go to great detail to discuss and differential whether biopsies were performed on native or transplant kidneys. However, I did not notice any discussion about whether patient perspectives were similar or different when they received a native or transplant biopsy. From a grounded theory approach, one would assume that these perspectives would be very different. I appreciate that this nuance may not emerge from a phenomenological approach, however, if it did emerge, it would be worth noting. If it did not emerge, please consider describing this in the limitations.

A2D: This qualitative study did not control for distinctions between the lived experience of individuals who undergo native and transplant biopsies, therefore a cautious approach is required in interpretation. Utilising a phenomenological approach in this small cohort, the lived experience of participants who had been previously transplanted at the time of their first biopsy may have been different to those who had not. There may be several reasons for this relating to the positioning of the patient, their previous experience of invasive procedures in the form of transplant surgery and familiarity with the clinical environment and staff. However further research would be needed to draw firm conclusions in this area.

Q2E: 5) Similarly, please provide justification for why the authors chose to use a phenomenological approach over a grounded theory approach. It is reasonable to pursue a phenomenological approach here, but discussing the rationale for choosing this approach would help strengthen the paper.

A2E: We agree with the reviewer that there are alternate qualitative research approaches to try and answer research questions.

A phenomenological approach was used to understand the direct ‘lived experiences’ of the patients undergoing a kidney biopsy i.e. we were interested in their own subjective understanding of having a kidney biopsy. There is a paucity of relevant literature in this area. If there were prohibiting circumstances that could prevent patients coming forward for biopsy, this could act as a barrier to timely diagnosis and treatment.

This explanation has been expanded in the manuscript for a more thorough justification of the methodological decision.

Minor comments:

Q2F: 1) In the background section, please provide more information on the incidence of kidney biopsies. If I were to just read the paper without any other clinical context, I'm not sure I would understand how big of a problem the authors are trying to tackle.

A2F: We agree entirely that this would help to provide context for the paper. Details of biopsy numbers within one region of the UK and internationally in terms of rates per million population annually have been added in the Introduction to the manuscript.

Q2G: 2) The authors discuss that the findings may have limited generalizability. As this is a qualitative study, please consider discussing transferability instead of generalizability.

A2G: Thank you for spotting this error. We have changed this to transferability.

Q2H: 3) The authors discuss how their work could help inform equitable access to care. However, it was not clear to me how the authors could link their work to health equity. I did not see a clear delineation of disparities in kidney biopsies, or how this patient perspective could address disparities. I do note that the interview guide asked for ethnicity of participants, however the body of the paper des not mention ethnicity. Please consider including ethnicity data. Please also consider adding a stronger rationale for how the study could potentially improve equitable access, or consider removing this section altogether.

A2H: This is an important point you have raised. Data on ethnicity has now been added. In terms of access to care, the authors do not propose that certain groups of individuals defined by age, sex or race are systematically disadvantaged with poorer access to kidney biopsy. We sought to examine the origins of patient reluctance or hesitation when this investigation was recommended by their healthcare provider. Specific patient-related factors may limit uptake, such as childcare provision to mothers of young children or loss of income for self-employed individuals. In our study, women reported specific challenges relating to urination whilst on bedrest during the observation period, which is a potential disparity that could be highlighted in advance for female patients. Understanding which patient-related barriers contribute to biopsy hesitancy allows for healthcare professionals to adapt services to help ensure individuals who are deemed to require this procedure can access it.

Reviewer #3: Toal et all carried out structure interviews with 11 patients who had undergone 23 kidney biopsies to get a sense of the themes of patient experience. The selection process was purposive, and the sample size, so no way to see if experience of participants who underwent native biopsy different from those who underwent transplant biopsies. A couple issues should be addressed.

Q3A: 1. Authors note the importance of knowing the person who performed the biopsy. But they note that MT as a physician may have influenced their results. How many of the biopsies did MT perform and how might that have influenced the interviews?

A3A: MT was not involved in the direct clinical care of any of the individuals who participated in the study at any point. Therefore, he performed none of the biopsies on these patients. MT has performed approximately 50 kidney biopsies across a five-year career in nephrology at the time of interview, however none of the patients he had biopsied were included in this study.

Q3B: 2. The psychological effects of the illness don’t seem a direct consequence of the biopsy but a consequence of the diagnosis that grew out of the biopsy. How do the authors tie this in with the biopsy.

A3B: This is an important distinction and has been highlighted by other reviewers. The kidney biopsy occurred within the context of each individual’s illness and the associated physical and emotional toll. We felt it was important to provide context to the reader that this invasive procedure was undertaken at a time when the participant was already vulnerable. The psychological effects may not have always been directly related to the procedure; however the biopsy was undertaken during the patient journey of living with their illness, therefore we felt it was important to allow participants to explain what this meant to them within that framework.

Q3C: 3. Can the authors speculate if there would be a difference between those who underwent native kidney biopsies and those who underwent allograft biopsies. Native biopsy patients have no idea what’s happening unless the operator tells them in advance since they’re lying on their stomach.

A3C: Within a qualitative research study with a small cohort, it is impossible to draw definite conclusions in this area, especially as we did not control for this variable, as it did not fall within the remit of the research question. However, there was some suggestion that the lived experience differed between these two groups. There may be several reasons for this relating to the positioning of the patient, their previous experience of invasive procedures in the form of transplant surgery and familiarity with the clinical environment and staff. In addition, the supine position for a transplant biopsy better facilitates communication between the patient and the operator. Further research would be required to explore these concepts in more detail.

Q3D: 4. Did any of the 10 suffered untoward consequences like bleeding, hematuria, hematoma after biopsy? How might the experience of those who experienced a complication differ from those who did not? If no one in the study group suffered a complication how generalizable are the findings to those patients?

A3D: Thank you for raising this point. As you have identified, a small sample size makes it difficult to capture the experiences of rare complications of biopsy. One participant reported hematuria and clots, which led to urinary retention. This man had undergone several biopsies, but understandably found that this procedure was the most difficult, because of the subsequent complication. The research team set out to understand the ‘lived experience’ of the typical kidney biopsy, as most patients do not have a serious complication. Further research is needed to identify persons who had a significant complication following biopsy and understand how this affected their perspective.

Additional amendment: On review of this manuscript, we discovered an error in the age at biopsy of one participant (P7). The age of first biopsy has been corrected from 13 to 17 years. The results section has been corrected accordingly.

6. PLOS authors have the option to publish the peer review history of their article ([what does this mean?](https://eur02.safelinks.protection.outlook.com/?url=https%3A%2F%2Fjournals.plos.org%2Fplosone%2Fs%2Feditorial-and-peer-review-process%23loc-peer-review-history&data=05%7C02%7Cmtoal11%40qub.ac.uk%7C23b1790c064e4e37b14c08dca1109f1b%7Ceaab77eab4a549e3a1e8d6dd23a1f286%7C0%7C0%7C638562341221623438%7CUnknown%7CTWFpbGZsb3d8eyJWIjoiMC4wLjAwMDAiLCJQIjoiV2luMzIiLCJBTiI6Ik1haWwiLCJXVCI6Mn0%3D%7C0%7C%7C%7C&sdata=3kC4RP25rpPGMrbThKZE%2BYaAU399tp4G5ehad2XyWhM%3D&reserved=0)). If published, this will include your full peer review and any attached files.


If you choose “no”, your identity will remain anonymous but your review may still be made public.


**Do you want your identity to be public for this peer review?** For information about this choice, including consent withdrawal, please see our [Privacy Policy](https://eur02.safelinks.protection.outlook.com/?url=https%3A%2F%2Fwww.plos.org%2Fprivacy-policy&data=05%7C02%7Cmtoal11%40qub.ac.uk%7C23b1790c064e4e37b14c08dca1109f1b%7Ceaab77eab4a549e3a1e8d6dd23a1f286%7C0%7C0%7C638562341221631144%7CUnknown%7CTWFpbGZsb3d8eyJWIjoiMC4wLjAwMDAiLCJQIjoiV2luMzIiLCJBTiI6Ik1haWwiLCJXVCI6Mn0%3D%7C0%7C%7C%7C&sdata=1qddaRzIKSu%2FI3ZuaXYnnqfxEO2QRoNHk87o8BJ%2B9%2Bc%3D&reserved=0).

Reviewer #1: No

Reviewer #2: No

Reviewer #3: **Yes:** George Bayliss, MD
